# Hyaluronic Acid Conjugates as Vectors for the Active Targeting of Drugs, Genes and Nanocomposites in Cancer Treatment

**DOI:** 10.3390/molecules19033193

**Published:** 2014-03-17

**Authors:** Silvia Arpicco, Paola Milla, Barbara Stella, Franco Dosio

**Affiliations:** Dipartimento di Scienza e Tecnologia del Farmaco (Department of Drug Science and Technology), University of Torino, Torino, I-10125, Italy

**Keywords:** bioconjugates, drug delivery, hyaluronic acid, anticancer agents, imaging agents, genes

## Abstract

Hyaluronic acid (HA) is a naturally-occurring glycosaminoglycan and a major component of the extracellular matrix. Low levels of the hyaluronic acid receptor CD44 are found on the surface of epithelial, hematopoietic, and neuronal cells; it is overexpressed in many cancer cells, and in particular in tumor-initiating cells. HA has recently attracted considerable interest in the field of developing drug delivery systems, having been used, as such or encapsulated in different types of nanoassembly, as ligand to prepare nano-platforms for actively targeting drugs, genes, and diagnostic agents. This review describes recent progress made with the several chemical strategies adopted to synthesize conjugates and prepare novel delivery systems with improved behaviors.

## 1. Introduction

Cancer is a leading cause of death worldwide, accounting for 7.6 million deaths (around 13% of all deaths) in 2008. Deaths from cancer worldwide are projected to continue rising, with an estimated 13.1 million deaths in 2030 [1,2]. Lung, stomach, liver, colon and breast cancers cause the most cancer deaths each year. 

Despite the enormous advances in understanding the molecular and cellular basis of cancer biology, and the development of improved therapies to treat malignancies, critical challenges remain in treating primary and metastatic disease, especially those located in the central nervous system, pancreas, and a number of other areas. Moreover, current anticancer drugs present some important drawbacks, including low specificity and high toxicity; these place serious limitations upon their efficacy. One of the most promising ways to create anti-cancer drugs with improved therapeutic index is based on targeted therapy; in recent years, significant progress has been made in developing molecularly-targeted cancer therapies.

In general, attempts to create tumor-selective cytotoxic drugs have focused on the physiological and biochemical differences existing between malignant and healthy tissues. It has been observed that, due to poor intra-tumoral lymphatic drainage and vascular leakage, large molecules can be made to preferentially accumulate at the malignant site: this phenomenon is known as the “enhanced permeability and retention” (EPR) effect [3]. In attempts to exploit this effect, polymeric conjugates [4], liposomes [5], microparticles, and nanoparticles [6] have been developed for possible use as passive drug delivery systems. Further, based on differences in biochemistry between cancerous and normal tissues, the same delivery systems have also been proposed to selectively target over-expressed tumor-specific receptors. 

Conjugation of cytotoxic drugs with macromolecules improves their pharmacokinetic profile, prolonging the distribution and elimination phases. Furthermore, the slow release of active drug from the carrier may result in sustained high intratumoral drug levels and lower plasma concentrations of the active drug. In order to achieve this combined effect, a macromolecule-drug conjugate should preferentially release the active drug within the tumor tissue. The following components are essential to reach this goal: a biodegradable linkage, a suitable spacer, and a potent bioactive anticancer agent. Among the most widely studied macromolecules are N-(2-hydroxypropyl) methacrylamide (HPMA), polyglutamate, human serum albumin, dextrans, heparin, chitosan, dendrimers, multi-arm polyethylene glycol (PEG), and hyaluronic acid [7,8,9]. To date, very few macromolecular drug conjugates have provided the dual advantages of accumulation at the tumor site and receptor-mediated uptake; hyaluronic acid (HA), a naturally-occurring polysaccharide, has the potential to do so. 

The molecular weight of native HA has a wide range. In water, high molecular weight HA self-aggregates to form a viscous solution in which each molecule forms a sponge-like matrix with a radius of about 100 nanometers; this makes it a suitable candidate for passive tumor accumulation. In addition, HA plays an important physiological role in the tumorigenesis process, and consequently HA receptors are overexpressed on many types of tumor cells [10]. This feature could be exploited in drug delivery, by using the receptor as an anchor to attach prodrugs or nanomedicine-based delivery systems, through a ligand, so as to increase the efficiency of anticancer drugs.

This review discusses recent progress and examines the various approaches that have been attempted to develop HA-based agents for cancer therapy and imaging, concentrating on those in which the active agents are covalently conjugated. For clarity’s sake, approaches employing HA as a formulation component, that is not bound to the drug or other active component, will not be examined. A rapid excursus from synthesis to pre-clinical/clinical evaluation of HA conjugates is included.

## 2. Hyaluronic Acid

**Figure 1:**
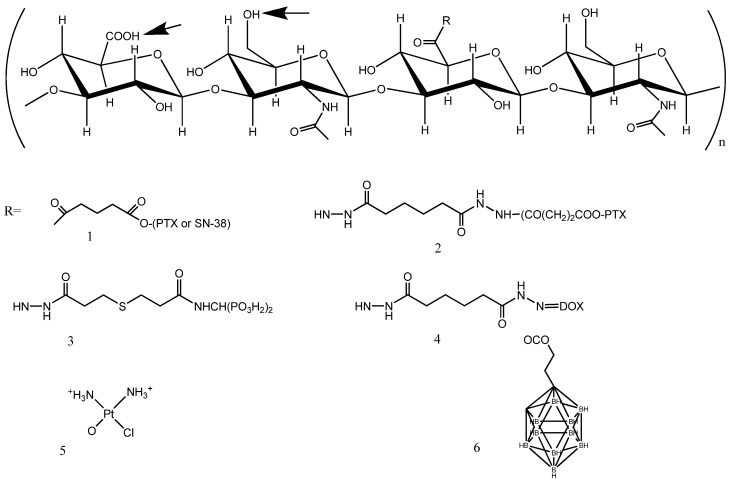
Hyaluronic acid and small molecule conjugates.

Hyaluronic acid is a high molecular weight (10^6^ –10^7^ Da) glycosaminoglycan polymer composed of repeating disaccharides: 𝛽1,3 N-acetyl glucosaminyl-𝛽 1,4 glucuronide (Figure 1). HA is ubiquitous, being the main component of extracellular matrix, and is essential for proper cell growth, structural stability of organs, and tissue organization. From the pharmaceutical standpoint, HA is a promising component, because it is biodegradable, biocompatible, nontoxic, hydrophilic, and nonimmunogenic. HA contains several chemical groups to which other components can be conjugated. The structure of HA is shown in Figure 1, where its possible chemical modification sites are also indicated (arrow). The carboxylate on the glucuronic acid, the N-acetylglucosamine hydroxyl, and the reducing termination, have all been successfully utilized in conjugation reactions with drugs. The acetyl group may be enzymatically removed from the N-acetylglucosamine, and is thus also a potential site for drug conjugation [11]. For a comprehensive review on chemical modification methods, synthetic routes to obtain HA derivatives, and their characterization, see Schanté *et al.* and Collins *et al.* [12,13].

In adult tissues, such as the vitreous humor, synovial fluid, and the dermis, hyaluronan plays an extracellular, structural role that depends both on its hydrodynamic properties and on its interactions with other extracellular matrix components. However, hyaluronan is also concentrated in regions of high cell division and invasion (during embryonic morphogenesis, inflammation, wound repair, and cancer). Hyaluronic acid is thus also involved in tumorigenesis.

Two HA receptors are strongly implicated in the cell signaling cascades associated with cancer initiation and progression: these are CD44 (cluster of differentiation 44) [14] and the receptor for hyaluronic acid-mediated motility (RHAMM) [15]. Furthermore, HA also interacts with ICAM-1 (intracellular adhesion molecule-1), TLR-4 (toll-like receptor-4), HARE (HA receptor for endocytosis), and LYVE-1 (lymphatic vessel endocytic receptor) [16]. The CD44 receptor, which belongs to the family of cell adhesion molecules (CAMs), is a widely-distributed transmembrane glycoprotein that plays a critical role in malignant cell activities, including adhesion, migration, invasion, and survival. 

CD44 mediates the internalization and metabolism of HA, and is endogenously expressed at low levels on various cell types in normal tissues [17], but it requires activation before it can bind to HA. Cellular activation can induce transition of CD44 to a high-affinity state, which is capable of binding HA. Transition from the inactive, low-affinity state to the active, high-affinity state of CD44 can be induced by ligation of antigen receptors [18], sulfation, or the action of cytokines [19]. Unlike normal primary cells, tumor-derived cells express CD44 in a high-affinity state, which is thus capable of binding and internalizing HA. Further, CD44 is reported to interact with HA of minimum length 6–8 saccharide units [20]. Interference with the CD44-HA interaction, by targeting drugs to CD44, targeting drugs to the HA matrix, or interfering with HA matrix-CD44 interactions, are possible strategies for cancer treatment. 

## 3. HA Drug-Conjugates

The earliest reports of an HA-drug conjugate designed to specifically target overexpressed CD44 were in a 1996 study on Lewis lung carcinoma cells by Akima *et al.*, which showed uptake of a fluorescent HA conjugate, of HA-conjugated mitomycin C (MMC), and of epirubicin. In a metastatic lung carcinoma model, HA-MMC decreased the number of metastatic lung nodules, thus increasing the therapeutic index, while HA-epirubicin showed no activity [21].

Again in 1996, two research groups, one from the pharmaceutical company Fidia, and the other coordinated by Prestwich, developed HA-butyrate and HA-paclitaxel. HA-butyrate, a histone deacetylase inhibitor, after conjugation showed increased apoptosis activity, inhibited cell growth *in vitro*, and resulted in a decreased tumor burden *in vivo* [22]. However, no further studies appeared after 2001. Conversely, studies on HA-paclitaxel have proven more promising, and this avenue has given rise to further studies.

### 3.1. HA-Paclitaxel

Paclitaxel (PTX) is a powerful drug recommended for ovarian, breast, lung, bladder, prostate, melanoma, esophageal, and other types of solid tumor cancers, as well as Kaposi's sarcoma [23]. However, PTX administration is problematic owing to its poor solubility and relevant side effects, and also due to the excipients typically used in its formulation; for these reasons, conjugation with HA may offer advantages. In an attempt to resolve these difficulties, HA (molecular weight ~200 kDa) was linked to PTX through 4-bromobutyric acid, generating two ester linkages between PTX and HA [HYTAD1-p20 (ONCOFID-P)] (Figure 1, R = 1) [24]. ONCOFID-P, with PTX loading of 20% w/w, was initially developed for treating superficial bladder cancer [24]. A subsequent imaging biodistribution analysis of a ^99^mTc-radiolabeled ONCOFID-P administered by i.v., i.p., intravesical, or oral routes was conducted [25]; i.v. injection was followed by rapid and marked liver uptake (around 80% of the injected dose). By contrast, imaging of the bladder, abdomen and gastrointestinal tract after administration showed that the radiolabeled conjugate remained compartmentally confined to the cavities. Thus these approaches may be relevant to locoregional treatment for transitional bladder cell carcinomas, ovarian cancers, and gastric tumors, respectively [25]. ONCOFID-P was subsequently evaluated for i.p. treatment of ovarian cancer against CD44 + OVCAR-3 and SKOV-3 human ovarian cancer cell lines, xenografted in nude mice [26,27]. ONCOFID-P cytotoxicity was somewhat lower than that of free PTX *in vitro*; however, i.p. treatment with ONCOFID-P was overall more effective than i.v. or i.p. administered PTX: it inhibited intra-abdominal tumor dissemination, abrogated ascites, prolonged survival, or even resulted in a cure for the test animals.

Currently, ONCOFID-P is in phase II marker lesion study in the intravesical therapy of patients with non-muscle invasive cancer of the bladder (EudraCT 2009-012274-13). Phase I studies have also been initiated to investigate the maximum tolerated dose and safety profile of ONCOFID-P following i.p. infusion, in patients affected by intraperitoneal carcinosis in ovarian, breast, stomach, bladder, or colon cancers [28,29,30].

The approach taken by Prestwich to deliver PTX with HA, from the chemical standpoint, involved creating a different, longer linkage between the two compounds; a succinate ester of adipic dihydrazide-modified HA (HA-ADH) was synthesized, followed by the coupling of PTX-N-hydroxysuccinimide ester (NHS) (Figure 1, R = 2). The HA used had a molecular weight of ~11 kDa [31]. *In vitro* and *in vivo* studies of a similar conjugate (HA 40 kDa) were carried out by Klostergaard and coworkers, on different cancers/cancer models; firstly, on a xenografted human ovarian carcinoma, by i.p. administration [32]; and secondly, on a human squamous cell carcinoma of the head and neck, by i.v. administration [33]. More recently, HA-ADH-PTX was administered i.p. with metronomic dosing, in a study that showed such regimens to have substantial antitumor activity in ovarian carcinoma, likely via a predominant antiangiogenic mechanism [34].

In an alternative strategy, described by Xin *et al.* PTX was conjugated onto HA (9.8 kDa) using carbodiimide via amino acid linkers; an amino acid (aa) was previously linked by its carboxylic group onto the 2' hydroxyl group of PTX. The intermediate was then conjugated by the amino group of the amino acid onto the carboxylic group of HA, using carbodiimide activation [35]. HA-aa-PTX prodrugs, loading 10%–15% w/w of PTX, self-assembled into nanoparticles with a size of 270–280 nm having neutral charge. *In vitro* data showed that cytotoxicity was higher than that of free drug.

Very recently, a conjugate between very low molecular weight HA (5 kDa) and PTX, through a direct ester linkage between 2' hydroxyl group of PTX and carboxylic acid of HA, was described [36]. To increase conjugate stability and solubility, the method described by Park [37] was applied: this involves the presence of dimethoxy-PEG (dm-PEG) to allow nanoparticle formation. Using this approach, two different populations of particles (2–3 nm and 80 nm) were observed. The conjugate, with a PTX loading dose of 8%, improved the standard chemotherapeutic drug’s efficacy in a preclinical model of brain metastases of breast cancer (231-Br cell line). The promising results on brain metastasis are apparently due to the small size of the nanoparticle conjugates, and to the ability of small HA conjugates to diffuse and accumulate in lesions better than larger ones, composed of higher molecular weight HA. In addition this approach also circumvents the blood brain barrier efflux transporter P-gp, enhancing cytotoxic activity inside the lesions. These data appear to be in agreement with other approaches to delivering PTX to brain tumors, for example those employing peptides, as in the case of GRN1005 [38].

### 3.2. HA Oncofid-S

The pharmaceutical company Fidia Co. has developed another promising conjugate of HA, named ONCOFID-S, using SN-38 (an active metabolite of irinotecan) (Figure 1, R = 1). The most promising results were achieved using a similar approach as for ONCOFID-P, with HA of 200 kDa and a derivatization degree of 9.5%. *In vitro* cytotoxicity was reported to be strong against several different CD44 + cell lines: colon adenocarcinoma, gastric, breast, esophageal, ovarian, and human lung cancer cell lines [39]. Pharmacokinetic evaluations lend support to the rationale for applying ONCOFID-S in the loco-regional intraperitoneal treatment of peritoneal carcinomatosis [40]. It is worth noting that formulations of HA with different anticancer drugs (irinotecan, doxorubicin, 5-fluorouracil) through HyACT^®^ technology (Alchemia), where the drug is not covalently linked to HA, also appear to be very promising. HA plus irinotecan is currently in phase III trials for colorectal cancer [41]. 

### 3.3. HA Bisphosphonates

**Figure 2:**
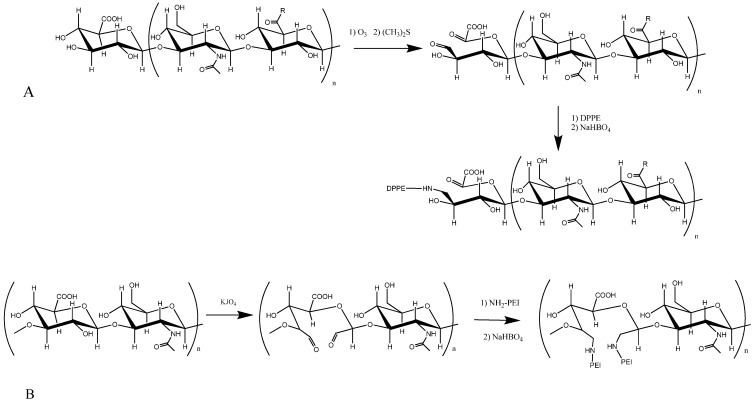
Synthetic scheme of hyaluronic acid-phospolipid- (**A**) and polyethyleneimine (**B**).

Third generation bisphosphonates (BP), such as zoledronic acid, exert a powerful antitumoral effect in a variety of human cancers [42]. With the aim of obtaining an antiosteoclastic and antineoplastic drug in an injectable hydrogel formulation, Varghese *et al.* synthesized a high-molecular-weight (HMW) HA (1.3 MDa) with different moieties. HA was functionalized on its carboxyl groups with both a hydrazide group and the bisphosphonate, by means of hydrazone linkages [43] (Figure 1, R = 3). The resulting compound was found to react with another proportion of HA, previously oxidized with periodate (as in Figure 2B), generating a hydrogel in less than 30 s. Cell internalization and cytotoxic properties of the HA compound were evaluated on two cell lines, which differed in terms of their cell surface HA receptor levels. The *in vitro* results showed that the HMW-HA-BP conjugate acted as a prodrug: HA was able to target CD44 +, but receptor-mediated endocytosis (and cytotoxicity) are only possible after cleavage of HMW-HA with a ubiquitous enzyme, hyaluronidase (Hase). In addition, the cytotoxicity of the HMW HA-BP conjugate was found to be directly proportional to cell surface HA receptor levels. The authors suggest that the hydrazide group of the HA-BP conjugate could also be used to explore hydrazone linkage of other drugs, such as doxorubicin (DOX), which could be integrated into the hydrogel matrix. Indeed, the synthesis and characterization of HA (150 kDa) DOX conjugates have very recently been reported [44] in research in which both a non-releasable amide (by direct linkage to DOX amine group) and an acid-sensitive hydrazone bond (through HA-ADH derivative, Figure 1, R = 4) were prepared; the DOX loading capacity was in the 0.2%–0.3% w/w range. DOX insertion caused a change in HA’s hydrophilicity profile, and self-assembly occurred, generating nanoparticles of 581–1600 nm. Preliminary cell toxicity data were reported. 

### 3.4. HA-Doxorubicin

In a study by Cai *et al.*, DOX and cisplatin were conjugated with HA but administered intralymphatically. Cisplatin was linked to the carboxyl groups of HA (35 kDa) using silver nitrate as activating agent; the optimal drug loading dose was 0.25% w/w (Figure 1, R = 5). The authors demonstrated that the intralymphatic delivery model, using the HA-cisplatin conjugate, not only significantly increased drug concentrations in loco-regional nodal tissues compared to the standard cisplatin formulation, but also exhibited sustained release kinetics [45]. A recent *in vivo* study on HA-cisplatin reported a significant improvement in antitumor efficacy, with lower toxicity compared to standard cisplatin, in locally-advanced head and neck squamous cell carcinoma [46]. In subsequent work, DOX was also reacted to HA (35 kDa) through a HA-ADH derivative, the optimal DOX loading degree being in the 5%–15% (w/w) range. Extended *in vivo* evaluations (renal and cardiac toxicity, pharmacokinetics, tumor model) were reported [47]. The peritumoral injection of HA-DOX led to efficient intralymphatic delivery that delayed tumor progression by approximately 10 weeks, and increased animals’ survival in comparison to i.v. free DOX treatment. Further studies demonstrated that subcutaneous nanocarrier delivery of doxorubicin and cisplatin possesses significant efficacy combined with decreased toxicity, achieving complete pathologic tumor response [48].

### 3.5. Boron Neutron Capture Therapy

Boron neutron capture therapy is a promising approach for tumor therapy [49], but appropriate targeting is required to achieve boron concentrations that are sufficient to deliver therapeutic doses of radiation to the tumor, with minimal normal tissue toxicity (~20 μg/g tumor). Di Meo *et al.* proposed a bioconjugate (HApCB) composed of n-propyl carborane, linked to HA via an ester linkage, for a degree of substitution of approximately 30%; the result was a water-soluble derivative (Figure 1, R = 6). The structural and physicochemical characteristics of HApCB were described and its cellular uptake was evaluated *in vitro* on a variety of human tumor cells; HApCB produced high intracellular accumulation of boron atoms [50]. More recently, the same group prepared and characterized two new HA derivatives bearing carborane rings: hyaluronan-amidoazido-carborane, and hyaluronan-propargylamido-carborane both caused relevant amounts of boron atoms to accumulate intracellularly in a variety of cancer cells [51]. The principal characteristics of HA-drug conjugates have been summarized in Table 1.

**Table 1:** Summary of the characteristics of the principal HA-drug conjugates.

HA Mw (kDa)	Drug	Conjugation chemistry	Name (or components)	DL	Particle size (nm)	*In vivo* admin.	Tumor model/clinics	Effects	Reference
200	Paclitaxel	Ester linkage	ONCOFID-P	20	--	IP, intravesical	Phase II bladder cancer	Prolonged survival (preclinical)	[24,25,26]
40	Paclitaxel	Ester linkage	HA-ADH+PTX-succ	15–20	--	IP, IV MTN	Ovarian cancer	Antiangiogenic	[31,32,33,34]
5	Paclitaxel	Ester linkage	+ dmPEG	8	--	IV	Brain metastasis. breast cancer	Inhibit tumor growth	[36]
200	SN-38	Ester linkage	ONCOFID-S	9.5	--	IP	Peritoneal carcinomatosis	Increase survival	[39,40,41]
13000	Aminomethylenediphosphonate	Hydrazone linkage	HA-hydrazone +HA-ox	5	--		CD44+ cells	Inhibition after Hyaluronidase	[43]
150	Doxorubicin	Acid-sensitive hydrazone or non-releasable amide linkage	HA-ADH or amide	0.2–0.3	581–1600		Different cell lines	Similar to DOX	[44]
35	Doxorubicin	Acid-sensitive hydrazone linkage	HA-ADH	5–15	--	SC	Xenografted human breast cancer	Better than IV DOX	[47]
35	Cisplatin	Cisplatin linked to the carboxyl groups of HA	HA-COO-Pt	0.25	--	SC	Xenografted human breast cancer	Better than IV cisplatin	[45]
200	Propyl-carborane	Ester linkage		30	--		Different cell lines	Uptake in CD44+ cells	[50,51]

HA, hyaluronic acid; ADH, adipic dihydrazide; DL, drug loading content (% w/w); MTN, metronomic therapy.

## 4. HA Decorated Particles

Gagomers (glycosaminoglycan cluster of particles; GAGs) are composed of lipid molecules that self-assemble into particulate clusters, which are then covalently coated with HMW (1.2–5 MDa) HA (by carbodiimide activation of carboxyl groups) at a lipid:HA ratio of 10:1 (w/w). HA is the main component of the particle surface; its interior contains both the lipid clusters and water regions, providing an intrinsic ability to accommodate both hydrophilic and hydrophobic molecules. GAGs can be generated as either micro-particles or nano-particles, and can encapsulate DNA, proteins, or small molecules with high efficiency. The HA coating naturally targets GAGs to cells expressing specific receptors, and gives these particles stealth properties.

The preparation of GAGs containing paclitaxel (PTX-GAGs) was first described by Rivkin *et al.*, who also reported the structural and physicochemical characterization of drug-free and PTX-loaded particles [52]. The cytotoxicity of PTX-GAGs was evaluated *in vitro* on the mouse colorectal carcinoma cell line CT-26, expressing CD44; toxicity was the same as that of free PTX. Pharmacokinetics, biodistribution and therapeutic properties of PTX-GAGs were evaluated *in vivo* on colon adenocarcinoma-bearing mice, and the results showed a good safety profile, marked tumor accumulation, and antitumor potency 4-fold that of Taxol^®^.

More recently, Bachar *et al.* tested drug-free GAGs *in vitro,* on primary head and neck cancers and normal cells taken from the same patient, and demonstrated that GAGs can bind selectively to the cancerous cells, although the CD44 expression did not differ significantly between normal and malignant cells. They prepared GAGs containing mitomycin C (MMC-GAGs) and tested their cytotoxicity *in vitro* on the same cell lines; the cytotoxicity of MMC-GAGs was significantly higher than that of free MMC on head and neck cancers, whereas the viability of normal cells was not affected [53].

### 4.1. Polymeric Nanoparticles

Several papers report HA conjugation to nanoparticles. With the aim of targeting 5-flurouracil (5-FU) to colon tumors, Jain *et al.* prepared chitosan–hyaluronic acid nanoparticles (HACTNPs) containing the drug. 5-FU loaded nanoparticles were prepared by the ionotropic gelation method, and their surface was coupled, using carbodiimide chemistry [1-ethyl-3-(3-dimethylaminopropyl) carbodiimide (EDC)], to the carboxylic group of HA, with chitosane amine groups forming an amide linkage. The structural and physicochemical characterization of HACTNP was described and its cellular uptake and cytotoxicity were evaluated *in vitro* on human colorectal adenocarcinoma HT-29 cell line, which over-expresses the CD44 receptor. The cellular uptake and cytotoxicity results suggest that the 5-FU loaded HACTNP formulation leads to increased uptake in comparison with HA-uncoupled chitosan nanoparticles (CNTPs), and that possesses enhanced cytotoxicity in HT29 colorectal cancer cell lines, compared with either CNTP or free 5-FU [54]. More recently, the same group prepared HACTNPs containing oxaliplatin, so as to investigate anticancer activity and biodistribution *in vivo* after oral administration in a murine colon-tumor model. The biodistribution studies indicated that the HACTNPs effectively reached the tumor, and were more effective in treating colon tumors compared with CNTPs or free oxaliplatin [55].

Nanoparticles have also been formed by covalent conjugation of HA to polylactide-co-glycolide (PLGA) via a PEG-2000 spacer. Both PLGA (50:50, MW 40–75 kDa) and HA (5.7 kDa) were first activated with EDC and NHS, then mixed with the diamine linker. The particles, whose size was in the 186 to 107 nm range, were loaded with DOX; they demonstrated increased effectiveness compared to nanoparticles of PLGA alone. Biodistribution studies showed high liver uptake, but also a significant concentration of HA-PEG-PLGA nanoparticles in tumor mass of Ehrlich ascites bearing mice [56]. A similar approach was employed to load 5FU [57], obtaining nanoparticles of 165 nm in diameter that demonstrated significant tumor uptake (4 h after administration) and thus anticancer activity.

Another interesting category of polymers suitable for drug delivery, poly(butyl cyanoacrylate) (PBCA), can be covalently derivatized with HA. In one such study, nanoparticles were obtained through radical emulsion polymerization of n-butyl cyanoacrylate monomers, initiated by cerium ions in the presence of HA. HA-PBCA nanoparticles entrapping PTX, having a diameter of 290 nm (best formulation HA/BCA ratios 1:2, HA 18 kDa) were found to be more potent in suppressing S-180 tumor growth than PTX-loaded PBCA nanoparticles or PTX injection [58].

HA was also conjugated via an ester linkage to ceramide (CE), a component of cellular membranes composed of sphingosine and fatty acid [59]. The amphiphilic conjugate was formulated with Pluronic 85 as nanoparticles to encapsulate docetaxel. *In vitro* studies showed that HA-CE nanoparticles enhanced the intracellular drug uptake in the CD44 + MCF-7 cell line. Moreover, the multidrug resistance-overcoming effects of these carriers were observed in cytotoxicity tests. *In vivo* studies after i.v. administration in mice of docetaxel-loaded HA-CE nanoparticles confirmed their targeting ability for CD44 + tumors. HA-CE conjugate was then linked to PEG via carboxyl groups of HA in order to increase the circulation time of the nanoparticles in the bloodstream [60]. PEGylation resulted in prolonged nanoparticle circulation and reduced drug clearance rate in an *in vivo* model.

HA was also directly conjugated to PLGA copolymer [61]. To this end aminated HA (obtained by reduction and further coupling to hexamethylene diamine) was reacted with NHS-activated PLGA and then the amphiphilic conjugate was used to form core-shell type nanoparticles containing DOX. Anticancer activity assays on CD44 + HCT-116 human colon carcinoma cells showed a selective uptake via receptor-mediated endocytosis. A similar approach was recently used to deliver docetaxel to CD44 + breast cancer [62]. In this study a deep investigation of HA-PLGA copolymers allowed nanoparticles to efficiently *in vivo* deliver docetaxel to breast cancer cells. HA was also grafted onto preformed PEG-PLGA nanoparticles by EDC coupling. The topoisomerase I inhibitor SN-38 was encapsulated to target ovarian cancer [63]. Cellular uptake and cytotoxicity of HA-PEG-PLGA nanoparticles were higher in CD44 + SKOV-3 and OVCAR-8 cell lines as compared to CD44- cells.

In another approach HA was chemically linked to 5β-cholanic acid and PEG to form tumor-targeted nanoparticles (P-HA-NPs); anticancer drugs (including DOX and camptothecin) were successfully encapsulated into the lipophilic inner core [64]. Since encouraging *in vitro* cell-specific activity results and *in vivo* tumor-targeting characteristics were obtained, further studies were performed in order to encapsulate several molecules, such as for simultaneous tumor-targeted photodynamic imaging and therapy with a hydrophobic photosensitizer chlorin e6 or a near-infrared fluorescence imaging dye plus irinotecan [65,66]. Moreover, to improve the *in vivo* stability of P-HA-NPs photo-crosslinked nanoparticles loaded with PTX were prepared via UV-triggered chemical crosslinking with acrylate groups in the polymer structure [67]. The release profile of PTX from nanoparticles was much slower than that from uncrosslinked P-HA-NPs. In addition, the photo-crosslinking procedure did not inhibited the *in vitro* CD44 receptor-mediated endocytosis and enhanced the *in vivo* targeting ability in tumor-bearing mice as a consequence of higher stability of the carriers.

An amphiphilic conjugate was obtained by reacting HA with glycyrrhetinic acid (GA), which is the metabolite of the natural product glycyrrhizin exhibiting several pharmacological activities, such as anti-inflammatory, immune-modulating and reversing the multidrug resistance to anticancer agents [68]. The HA-GA conjugate was formulated as PTX-loaded nanoparticles actively targeted towards liver tumor. HA-GA carriers showed a higher cytotoxicity on cells that overexpress both HA and GA receptors. *In vivo* assays showed an accumulation in tumor and liver of mice.

An interesting targeted codelivery system was obtained from an amphiphilic HA-all-trans retinoid acid (HRA) conjugate forming nanoparticles for potentially synergistic combination chemotherapy of paclitaxel and retinoic acid [69]. The HRA conjugate was obtained by reacting aminated retinoid acid with HA carboxyl groups. PTX-loaded HRA nanoparticles showed greater *in vitro* and *in vivo* anticancer activity.

### 4.2. Microcarriers

Hyaluronic acid can also be conjugated to microparticles. A first attempt in this connection aimed to obtain a cisplatin carrier that could attach itself to tumor cells, once it had reached the tumor nodules on the peritoneal surface, after i.p. administration [70]. The microparticles were composed of cisplatin linked to the carboxylic groups of HA, which served as a biodegradable and biocompatible polymeric matrix, onto which to link the drug (27.3% drug content, w/w) and at the same time as a targeting agent for the CD44 receptor. It emerged that this particulate system had favorable pharmacokinetic characteristics, and was more effective than the parent drug in inhibiting the growth of ovarian cancer nodules.

In another approach, HA was first oxidized to obtain aldehyde groups, and then linked via an acetylation coupling to poly(vinyl alcohol) (PVA), a polymer already used in biomedicine as bioinert support material [71]. In particular, PVA formed the shell of microbubbles that had an air-filled core, and were used as ultrasound contrast agents; this particulate system, conjugated to oxidized HA, was characterized. DOX was also loaded into PVA-shelled microbubbles, and liquid-filled PVA microcapsules were derived, with the goal of obtaining a new theranostic tool. Analysis of the *in vitro* behavior of these microcarriers showed that the HA-based coating played a key role in promoting the microparticles’ internalization into a CD44 + HT-29 cell line. Table 2 shows the principal characteristics of HA-nano and microparticle conjugates described above.

### 4.3. Liposomes and Lipoplexes for Delivering Antitumoral Drugs, DNA and RNA

HA has successfully been linked to liposomes following two different conjugation strategies. In the first method, HMW HA was linked via the glucuronic carboxylate to the amino reactive group of phosphatidylethanolamine (PE) on the surface of preformed liposomes. This amidation reaction was carried out after preactivating HA with a water-soluble condensing agent (EDC), by adding the activated HA to the liposome suspension [72,73,74].

**Table 2:** Summary of the characteristics of the principal HA-nanoparticle and microparticle conjugates.

HA Mw (kDa)	Drug	Conjugation chemistry	Name (or components)	EE (LD)	Particle size (nm)	*In vivo* admin.	Tumor model	Effects	Ref.
500–1200	Paclitaxel	Carbodiimide conjugation	PTX-GAG	100	316 ± 23	IV	Colon adenocarcinoma-bearing mice	Antitumor potency 4-fold higher than Taxol®	[52]
750	Mitomycin C	Carbodiimide conjugation	MMC-GAG	68–97	350 ± 35		Head and neck cancers cells	Higher cytotoxicity compared with free MMC	[53]
15000	Oxaliplatin	Carbodiimide conjugation	Oxaliplatin-HACTNP	40	152 ± 5.2	Oral	Murine model, colon tumor	Higher antitumor potency compared with free drug	[55]
5.7	Doxorubicin	HA linked to PLGA via a diamine PEG spacer	DOX-HA-PEG-PLGA NP	90	186–107	IV	Ehrlich ascites bearing mice	High tumor uptake, reduction of tumor size	[56]
5.7	5-flurouracil	HA linked to PLGA via a diamine PEG spacer	5FU-HA-PEG-PLGA NP	80	165	IV	Ehrlich ascites bearing mice	High tumor uptake, reduction of tumor size	[57]
18	Paclitaxel	Radical polymerization of HA and BCA monomers	PTX-HA-PBCA NP	90	290	IV	Sarcoma bearing mice	High tumor uptake, Reduction of tumor size	[58]
4.7	Docetaxel	Ester linkage	HA-CE-DOC	72 (11)	111	IV	MCF-7/ADR tumor-bearing mice	Reduction of tumor size *in vivo*	[59]
4.7	Docetaxel	Ester linkage	HA-CE-PEG-DOC	91 (12)	160	IV	Squamous cell carcinoma mouse model	Reduction of tumor size.	[60]
7.5	Doxorubicin	Amide linkage	HA-PLGA-DOX	68 (8)	72 ± 21		Human colon carcinoma cells	More efficient DOX internalization in CD44+ cells	[61]
5.6, 7.3 or 8.9	Docetaxel	Amide linkage	HA-PLGA-DOC	88 (3)	117	IV	Human breast tumor-bearing mice	Enhanced antitumor activity compared with free drug	[62]
234	Camptothecin	Amide linkage	CPT-P-HA-NP	86 (34)	320 ± 13	IV	Ovarian carcinoma-bearing mice	Specific tumor accumulation	[64]
234	Chlorin e6	Amide linkage	Ce6-HA-NP	62 (12)	227 ± 12	IV	Human colon cancer-bearing mice	Increased antitumor efficacy	[65]
234	NIR-fluorescence dye	Amide linkage	Cy5.5-P-HA-NP	--	237–424	IV	Human colon cancer-bearing mice	Specific tumor accumulation	[66]
234	Irinotecan	Amide linkage	IRT-P-HA-NP	62 (19)	238 ± 7	IV	Human colon cancer-bearing mice	Higher antitumor activity and reduction of systemic toxicity	[66]
10	Paclitaxel	Amide linkage	HA-GA-PTX	92 (31)	321 ± 2.5	IV	Human breast carcinoma	Specific tumor accumulation	[68]
10	Paclitaxel	Amide linkage	PTX-loaded HRA	91 (29)	149 ± 10	IV	Subcutaneous melanoma	Specific tumor accumulation	[69]
600–1200	Cisplatin	Cisplatin linked to the carboxyl groups of HA	HA-Cisplatin-Microparticles	50	580	IP	Ovarian cancer tumor-bearing mice	Slowing the growth of tumor	[70]
700	Doxorubicin	Interfacial acetalization reaction	HA-PVA-DOX Microparticles	--	--		Human colon cancer-bearing mice	The effect of DOXO within the first 3 days is cytostatic	[71]

EE, encapsulation efficacy (%); LD, drug loading content (w/w%); GAG, glycosaminoglycan cluster of particles; HACTNPs, chitosan–hyaluronic acid nanoparticles; PLGA, polylactide-co-glycolide; PBCA, poly(butyl cyanoacrylate); CE, ceramide; HRA, HA-all-trans retinoid acid; PVA, poly(vinyl alcohol); MCF-7/ADR, breast cancer adriamycin resistant line.

This approach offers the advantage that HA is only conjugated on the particle surface; however, the reaction leads to multipoint attachment of the polymer on the liposomes, and consequently the density of attachment is difficult to control and to characterize.

HMW-HA was used, in studies by Peer et al [73], to decorate liposomes encapsulating anticancer drugs. In a first study, MMC was encapsulated in HA-liposomes, and their *in vitro* and *in vivo* behavior was compared to that of plain liposomes. The cytotoxic activity of the drug encapsulated into HA-liposomes was found to be 100-fold that of free drug, in tumor cell lines overexpressing the HA receptors, but not in cells with low receptor expression levels. *In vivo* studies confirmed the higher antitumor activity of HA-liposomes containing MMC over free drug or unmodified liposomes. The same group obtained similar results with DOX encapsulated in liposomes, on different tumor models, confirming the important and efficient targetability of HMW-HA as vector [72]. These studies demonstrate the advantages of HMW-HA as targeting agent for liposomes, namely: its high binding affinity for the CD44 receptors, its ability to provide long-term circulation *in vivo*, and its ability to act as cryoprotectant.

In the second method, a conjugate between HA and an aminoreactive group of a lipid was prepared and purified, and then added to the lipid mixture during liposome preparation. With this approach, two different conjugation methods have been developed, depending on the molecular weight of HA. Low molecular weight (LMW) HA, obtained either by enzymatic degradation or from commercial sources, is linked to PE by reductive amination, using sodium cyanoborohydride as reducing agent, and giving a conjugate in which only one PE molecule is linked to HA [75,76]. This procedure enables a controlled amount of HA to be introduced into the liposomes, but may require more elaborate synthesis.

The targetability of LMW-HA decorated liposomes was first demonstrated by Eliaz and Szoka [75], who attached HA oligosaccharides to PE, subsequently incorporating the conjugate into the liposomes. HA was found to increase the liposomes’ recognition by B16F10 cells expressing high levels of CD44 in a temperature-dependent manner; uptake of the liposomes was also dependent on the density of HA oligosaccharide on their surface. Conversely, the control CV-1 cells, with low CD44 levels, showed little HA-liposome uptake. Moreover, encapsulated doxorubicin was more efficient at killing B16F10 cells than was free drug, indicating that these HA-liposomes may provide an efficient drug delivery system to CD44 expressing cells. A similar approach was used by our group, to prepare HA-liposomes containing a gemcitabine prodrug for active targeting against pancreatic cancer cells [77,78]. Conjugates between 1,2-dipalmitoyl-*sn-*glycero-3-phosphoethanolamine (DPPE) and two different LMW-HA (4.8 kDa and 12 kDa) were prepared, characterized, and introduced into liposomes during their preparation. *In vitro* studies demonstrated that HA facilitates the recognition of liposomes by MiaPaCa2 cells (CD44 +) and that the uptake increases as the polymer’s molecular weight increases; moreover, HA-liposomes also inhibited cell growth more than plain liposomes. *In vivo* studies showed that all the liposomes possessed higher antitumoral activity than free drug, in a mouse xenograft tumor model of human pancreatic adenocarcinoma. In particular, the 12 kDa HA-liposomes showed the strongest efficiency, while plain liposomes and the 4.8 kDa HA-liposomes had a similar activity. 

Cho *et al.* applied the above described HA-ceramide conjugate strategy [59] for the preparation of nanohybrid liposomes coated with HA-CE, for the targeted delivery of DOX and of a contrast agent for magnetic resonance imaging (Magnevist) for *in vivo* cancer imaging. The *in vitro* results showed the drug’s cellular uptake from nanohybrid liposomes to be higher than that of plain liposomes enhanced by HA and CD44 receptor interaction. *In vivo* contrast-enhancing effects revealed that the nanohybrid liposome can be used as a tumor targeting magnetic resonance imaging probe for cancer diagnosis. Pharmacokinetic studies showed prolonged circulation of nanohybrid liposomes *in vivo* [79].

HMW-HA was used by our group to prepare conjugates with the fusogenic phospholipid DOPE (dioleoylphosphatidylethanolamine), by means of an amidation reaction [80,81,82] (Figure 2A). In this conjugate, the DOPE amino group is randomly linked to the carboxylic residues of the polymer, and then introduced into the cationic lipid mixture during lipoplex preparation [80]. This kind of conjugate can be used to deliver plasmid DNA and siRNA to CD44 + cancer cells [80,81,82]. The presence of HMW-HA in the lipoplexes was found to enhance nucleic acid protection from degradation against DNase I or RNAse VI. The HA-DOPE conjugate increased the transfection efficiency on MDA-MB-231 or A549 cell lines, with high CD44 levels, compared to its effect on cell lines expressing low levels of CD44 (MCF-7 or Calu-3). Moreover, the transfection was markedly inhibited by using the anti-CD44 Hermes-1 antibody, indicating specific uptake of the lipoplexes through the CD44 receptor.

### 4.4. Polyethyleneimine

Polyethyleneimine (PEI) is one of the most widely used cationic polymer non-viral vectors [83]. PEI is very efficient, highly cytotoxic, due to an excess of positive charges; conjugation with HA has enabled carriers to be prepared with the target-specificity and biocompatibility of HA, and with a partial block of the positive charges, but that are still able to act as non-viral nucleic acid carrier [84,85,86].

Both HMW and LMW-HA have been used to prepare PEI-HA conjugates. When HMW-HA is used, the HA carboxyl groups are preactivated and then conjugated to the amines of PEI via an amide bond [87,88]. In these conjugates involving HMW-HA, the range of PEI:HA mass ratios explored has involved a larger amount of PEI than of HA within the system. The other main approach to prepare PEI-HA conjugates uses LMW-HA, and the synthetic method is based on reacting the primary amines of PEI to one of the terminal ends of the HA chain, via a reductive amination process [89]. These conjugates are characterized by a higher content of HA than of PEI.

Jiang, *et al.* developed a novel target-specific siRNA delivery system, using PEI conjugated to HMW-HA [90]. The conjugates were able to efficiently complex siRNA by means of electrostatic interactions with the amino groups of PEI. The study demonstrated HA-specific binding *in vitro*, reporting preferred uptake and enhanced silencing effects in B16F1 cells than in HEK 293 cells, which do not express HA-specific receptors.

Yao *et al.* synthesized a PEI-HA copolymer for targeted gene delivery, prepared by an imine reaction between periodate-oxidized HA of different molecular weights (60 kDa, 500 kDa, and 1500 kDa) and PEI (Figure 2B). Compared to the PEI/DNA complex, the PEI-HA conjugates showed an improved ability to form complexes with DNA, better physicochemical properties, and lower cytotoxicity, and they also exhibited higher transfection efficiency in HepG2 cells. The effect of HA’s molecular weight was also evaluated, showing that the complex prepared using HA of molecular weight 500 kDa was the most efficient. Moreover, the complexes that contained HA-PEI accumulated to a greater extent in tumor tissues after i.v. administration, compared to PEI, showing that PEI-HA could facilitate DNA targeting to the tumor [91]. Needham *et al.* conjugated PEI with HA oligosaccharides of different lengths, and after evaluating their ability to transfect human mesenchymal stem cells, they suggested that HA containing 10 saccharide units was sufficient to enhance transfection efficiency [92]. A (PEI-SS)-HA conjugate has also been developed, as a target-specific and non-toxic delivery system for siRNA. PEI-SS was prepared by cross-linking PEI with cystamine bisacrylamide, and further conjugation with LMW-HA by reductive amination. The study demonstrated an enhanced therapeutic effect of the siVEGF/SS-PEI-HA-nanoparticles after intratumoral injection, compared to siVEGF/SS-PEI-particles, indicating that tumor-specific targeting had been conserved [93]. The same group recently described the preparation of a reducible HA−siRNA conjugate for target-specific systemic delivery of siRNA to the liver. The HA−siRNA conjugate was prepared through a disulfide exchange reaction between HA-SPDP and siRNA-SH. The conjugate was then complexed with PEI to form stable nanocomplexes. The results showed that conjugation of siRNA to HA enhances the resistance to RNase and facilitates receptor-mediated endocytosis. Good *in vitro* gene-silencing efficiency of the HA−siRNA/PEI complex was observed; *in vivo* experiments showed, further, that the complex was specifically and efficiently delivered to the liver [94].

### 4.5. Polymersomes

Polymer vesicles (polymersomes) based on poly(γ-benzyl l-glutamate)-block-hyaluronan produced by click chemistry have been recently reported [95]. In this kind of system, the hyaluronan block favors both colloidal stability of the vesicles, by means of electrosteric effects, and interaction with CD44 hyaluronan receptors. DOX-loaded polymersomes have been found to efficiently encapsulate and deliver the drug intracellularly, in CD44 receptor-expressing C6 glioma cells *in vitro*. The cytotoxicity of these polymersomes loaded with DOX or docetaxel was determined *in vitro* in cancer cell lines expressing different levels of CD44 receptors, such as the human glioblastoma cell line (U87) and the human breast cancer cell line MCF7 [96]. Blood clearance and biodistribution profile studies, in CD44 + expressing Ehrlich Ascites Tumor (EAT) bearing Balb/C mice, were then performed on drug-loaded polymersomes labeled with ^99m^Tc. Biodistribution data demonstrated that polymersomes accumulated at the tumor site, due both to passive accumulation (EPR effect) and to active targeting (CD44 mediated endocytosis) in EAT-bearing mice. Polymersome uptake in the tumor was greater at all time points, compared to free drug [97,98].

### 4.6. Micelles

The hydrophilic backbone of HA can be conjugated to hydrophobic portions, giving amphiphilic macromolecules that can form micelles. In recent work, HA was linked via its carboxylic groups to amino functions of poly(l-histidine) (PHis), forming a tumor-targeted copolymer (HA-PHis) that can self-organize into micelles and carry anticancer drugs, such as DOX [99]. Moreover, the resulting HA-PHis copolymer contained pH-responsive blocks in its structure, enabling DOX to be released *in vitro* in a pH-dependent manner, exploiting the lower pH of cancer cells. In a prior study by the same group, another stimuli-responsive micelle material was prepared by adding an amphiphilic moiety (deoxycholic acid, DOCA) to HA [100]. In particular, cystamine was coupled via an amidic linkage to HA and DOCA and it was exploited as a bioreducible linkage to prepare redox-sensitive HA-deoxycholic acid (HA-ss-DOCA) PTX-loaded micelles. The degradation of these carriers depends on the glutathione concentration, which acts on the disulfide bond; drug-loading capacity for PTX was excellent, and anticancer activity both *in vitro* and *in vivo* was higher than that of insensitive micelles (HA-DOCA carriers lacking the disulfide bond). PTX has also been successfully encapsulated into micelles characterized by a dual targeting strategy: folic acid (FA) was conjugated to HA linked to hydrophobic octadecyl moieties (FA-HA-C_18_) to give self-assembling compounds [101]. In particular, grafting of the octadecyl chains exploited the reaction between the carboxyl group of HA and the amine of octadecylamine, while FA was linked via an ester bond to the hydroxyl groups of HA. The dual targeting resulted in excellent uptake into cancer cells. PTX has also been directly conjugated to HA via an ester linkage in a single organic phase, to form nanosized micellar aggregates that release the drug at acidic pH [37]. These HA-PTX micelles are more cytotoxic for HA receptor-overexpressing cancer cells than they are for HA-receptor negative cells.

The grafting of HA to biodegradable copolymers to form DOX-loaded micelles has been reported. In the first such study, HA was conjugated to hydrophobic Poly[lactic-*co*-(glycolic acid)] (PLGA) using the PEG-assisted solubilization method in anhydrous DMSO; the resulting amphiphilic copolymer self-assembled to form multi-cored micellar aggregates [102]. DOX was entrapped during micelle formation, and the carriers exhibited higher cellular uptake and greater cytotoxicity on CD44 + than free drug. In another study, HA was grafted onto hydrophobic polylactic acid (PLA) and amphiphilic PEG-PLA copolymers, to obtain a hydrophobic/hydrophilic balance suitable to form DOX-loaded polymeric micelles in aqueous medium [103]. PEGylated micelles, which exhibited greater stability and had a higher drug loading capacity, were less sensitive to the adsorption of proteins from the cultured medium. Moreover, as shown by cytotoxicity tests on PEGylated and non-PEGylated micelles, the presence of PEG on the carrier’s surface did not interfere with CD44 receptor recognition.

HA has also been used to prepare diagnostic nanocomposite tools, as will be discussed in some depth below. In particular, magnetic nanoclusters were modified with HA (HA-MNCs) in a study aimed at detecting CD44 + breast cancer by magnetic resonance imaging [104]. To increase HA’s hydrophobicity, it was conjugated to 1-pyrenylbutyric acid, giving CD44-targetable surfactants that were then formulated with hydrophobic magnetic nanocrystals using the nano-emulsion method. HA-MNCs exhibited superior biocompatibility and excellent properties for the targeted diagnosis of CD44-overexpressing breast cancer *in vitro* and *in vivo*. Table 3 summarizes the principal characteristics of HA-conjugates reported in section 4.3 to section 4.6. 

## 5. Theranostic Nanoparticles Conjugated with HA

The match between nanotechnology and oncology has given rise to a new field of interdisciplinary research, and several multifunctional nanocompounds have recently been developed that combine therapeutic and diagnostic properties. Theranostic nanomedicines, which include quantum dots, carbon nanotubes and nanodots, graphene, gold nanoparticles, iron oxide nanoparticles, and silica nanoparticles, have been found to acquire novel characteristics after their conjugation with HA. 

**Table 3:** Summary of the characteristics of the principal HA-liposome, polymersome, micelle and lipoplex conjugates.

HA Mw (kDa)	Drug	Conjugation chemistry	Name (or components)	EE (LD)	Particle size (nm)	*In vivo* admin.	Tumor model	Effects	Ref.
Nr	Mitomycin C	Amidation reaction	HMW-HA-lip-MMC	53	--	IV	Different human models in mice	High tumor uptake, reduction of tumor size	[72]
Nr	Doxorubicin	Amidation reaction	HMW-HA-lip-DOX	78	81 ± 13	IV	Different human models in mice	High tumor uptake, reduction of tumor size	[73]
0.8–3	Doxorubicin	Reductive amination	LMW-HA-lip-DOX	90	110–140		Murine melanoma cell line	Higher cytotoxicity compared with free DOX	[75,76]
4.8–12	Gemcitabine prodrug	Reductive amination	LMW-HA-lip-Gem prodrug	89	154–192	SC	Pancreatic adenocarcinoma cell line	Higher cytotoxicity compared with free gemcitabine	[77,78]
4.7	Doxorubicin	Ester linkage	HACE-DOX-Magnevist	59 (1.6)	125 ± 5	IV	Human breast tumor--bearing mice	High tumor uptake and DOX prolonged circulation	[79]
1500	pDNA	Amidation reaction	HMW-HA-DOPE-pDNA		250–350		Human breast tumor cell line	Transfection of pDNA into MDA-MB-231 cells	[80,81]
1500	siRNA	Amidation reaction	HMW-HA-DOPE-siRNA		200		Lung cancer	Transfection of siRNA into A549 cells	[82]
130	siRNA	Amide linkage	HA-PEI-siRNA		21		Murine melanoma	Transfection of siRNA into B16F1 cells	[90]
60–500–1500	DNA	Amide linkage	HA-PEI-DNA		200	IV	Hepatocellular carcinoma	Transfection of DNA into HepG2 cells; high tumor uptake *in vivo*	[91]
6.7	siRNA	Reducible SS bond	HA-PEI-SS-siRNA		110	intratumoral	Murine colorectal tumor	Inhibited tumor growth with reduced VEGF mRNA and VEGF levels in the tumors	[92,93]
5.14	Doxorubicin	Huisgen 1,3-dipolar cycloaddition (“Click” chemistry coupling)	HA-poly(g-benzyl l-glutamate)-DOX	50 (12)	220	IV	Rat breast carcinoma model	Higher tumor uptake, higher tumor suppression and	[96]
5.14	Docetaxel	Huisgen 1,3-dipolar cycloaddition (“Click” chemistry coupling)	HA-poly(g-benzyl l-glutamate)-DOC	49 (10)	135 ± 10	IV	Ehrlich Ascites Tumor	Equipotent or more potent than free DOC	[97,98]
11	Doxorubicin	pH responsive linkage	HA-PHis-DOX (pH responsive)	85–91 (4–6)	155–215		Human breast tumor cell line	High cytotoxicity	[99]
11	Paclitaxel	Reducible SS bond	HA-SS-DOCA-PTX	93 (34)	119 ± 5		Human breast tumor-bearing mice	tumor accumulation *in vivo*	[100]
250	Paclitaxel	Ester linkage	FA-HA-C_18_-PTX	97 (9)	206 ± 14		Human breast tumor cell line	Higher cytotoxicity than Taxol	[101]
17	Doxorubicin	Ester linkage	HA-PLGA-DOX	31 (7)	119 ± 3		Human colorectal carcinoma	Greater cytotoxicity than free DOX	[102]
221	Doxorubicin	Ester linkage	HA-PLA-DOX	10 (5)	41 ± 1.5		Human colorectal carcinoma and fibroblasts	Less cytotoxic than free DOX	[103]
221	Doxorubicin	Ester linkage	HA-PLA-PEG-DOX	20 (10)	37 ± 0.8		Human colorectal carcinoma and fibroblasts	Less cytotoxic than free DOX	[103]
1000	magnetic nanocrystals	Amide linkage	HA-MNC	78	72–138	IV	Human breast tumor-bearing mice	Specific tumor uptake (magnetic resonance imaging)	[104]

Nr, not reported; EE, encapsulation efficacy (%); LD, drug loading content (w/w%); PLA, polylactic acid; MNC, magnetic nanoclusters.

### 5.1. Quantum Dots

Quantum dots (QDs) are nanocrystals made of semiconductor materials. They have unique light-emitting properties that can be optimized by tuning their size and composition. QDs are made up of CdTe/CdSe, Cd_3_P_2_, InAs/ZnSe, and InAs/InP/ZnSe, and characteristically, they possess enhanced quantum photoluminiscent properties. Coating with ZnSe enhances the efficiency of QD nanoparticles. In order to achieve stable and improved molecular imaging, a coating around the QDs is applied using various ligands or organic compounds [105]; however, interaction between the coating agent and the QDs may lead to photoinstability [106]. Conversely, their inherent toxicity (because they release cadmium, although at low concentrations) restricts their possible biological applications, and several coating approaches are under study.

**Figure 3:**
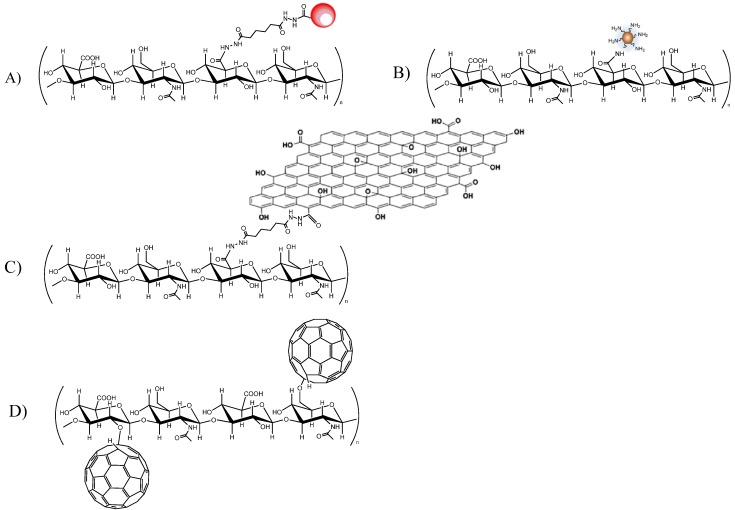
Quantum dots (**A**), carbon nanodots (**B**), graphene oxide (**C**), fullerene (**D**) conjugates with HA.

Hahn’s group has reported some interesting results on QDs coated with HA. The conjugation process that they have studied involves linkage of adipic acid dihydrazide-modified HA (HA-ADH) onto QDs having carboxyl terminal ligands, which were activated with EDC and N-hydroxysulfosuccinimide (sulfo-NHS) [87,107] (Figure 3, A). QDs with an emission wavelength of 650 and 800 nm were tested. Using HA of 130 kDa, it was found that HA-QDs conjugates with 35 mol% ADH content, which maintain sufficient binding sites for HA receptors, mainly accumulated in the liver, whereas those with 68 mol% ADH content, which lose many characteristics of HA, were evenly distributed throughout the body tissues. Furthermore, these data were confirmed in later research, in which QDs with HA modified with 22 mol % ADH were submitted to real-time bio-imaging on normal and cirrhotic mice [108]. HA-QDs conjugates were delivered in a target-specific manner, and accumulated more efficiently in the cirrhotic liver, where they remained even after 8 days. Slightly modified HA–QDs conjugates, which still had adequate binding affinity to HA receptors, mainly accumulated in the liver, whereas highly modified HA–QDs conjugates were evenly distributed throughout the body, exactly as were synthetic PEGylated QDs. 

The same approach was also employed to generate conjugates of HA with DOX; this conjugate then self-assembled to form micelle-like nanoparticles. To enhance their applications, the micelles were coated with a gold half-shell, by the thermal vapor deposition method; this may contribute to prolonging the residence time in the body [109]. 

### 5.2. Carbon-Based Nanostructures

In recent years, a family of carbon structures, including fullerene [110], carbon nanodots [111], carbon nanotubes (CNTs) [112], graphene [113] and graphene quantum dots [114], have attracted very considerable research interest, due to their superior and interesting properties, and some trial applications have been tested. The unique properties of nano-carbon based materials depend notably on their material dimensionality. For example, graphene QDs act as zero-dimensional materials, CNTs as one-dimensional (1D) materials, and graphene sheets as ideal two-dimensional (2D) materials. The inert composition of these materials is greatly beneficial, because of their biocompatible behavior, which is highly desirable for biological applications. In order to improve their characteristics of solubility and targeting, novel carbon materials have been investigated for therapeutic and bio-imaging applications, after conjugation with HA. Extensive research has been carried out in this field by Korean scientists, and almost all carbon-based nanostructures are linked to HA. 

#### 5.2.1. Carbon Nanodots

Carbon nanodots (Cdots) have many advantages, such as chemical inertness, lack of blinking, inherently low cytotoxicity, excellent biocompatibility, size- and excitation-wavelength dependent photoluminescence, and amphiphilic characteristics depending on the surface capping materials [115] Cdots have been prepared by the pyrolysis of citric acid in the presence of PEG diamine (Mw 2,000) as a capping material. HA Cdot conjugates were synthesized by amide bond formation between carboxyl groups of HA (100 kDa) and amine groups of Cdot−PEG-amine, using the carbodiimide reaction (EDC) (Figure 3, B). The resulting HA−Cdot conjugates had a mean particle size of 68 nm. The *in vivo* real-time bio-imaging of Cdots and HA−Cdot conjugates, in Balb/c mice, confirmed the target-specific delivery of HA conjugate to the liver; this is due to the abundant HARE and CD44 receptors in that organ. Low fluorescence intensity was observed for Cdots, which might reflect the rapid renal clearance of Cdots having a particle size in the 5–7 nm range [116].

#### 5.2.2. Graphenes

Very recently, graphenes and their derivatives, as single-layered carbon materials, have attracted considerable interest from biomedical researchers. Their unique 2-D structure provides a large surface area on both sides of the sheet, for the physical adsorption of nucleobases and aromatic compounds, mainly through π–π stacking; the functional groups, including epoxy, hydroxyl, and carboxylic acid moieties, that are attached to graphene oxide (GO) sheets facilitate their conjugation with biomolecules. Graphenes have additional advantages, including NIR photoluminescence for bioimaging, and photothermal effects for cancer therapy [117].

Li *et al.* [118] employed HA conjugated GO in photodynamic therapy studies. The GO was linked with oligo HA 5.8 kDa, by reaction with ADH and conjugation with EDC (Figure 3, C). Nanohybrids of 78 nm average diameter, and with about 18% of GO sheet in the HA–GO conjugate, were obtained.

HA–GO had excellent solubility and colloidal stability, qualities that are provided by the HA chains. Physical loading with the photodynamic agent (chlorin e6; Ce6) was then achieved by simple dialysis, obtaining a loading content of Ce6 of 115%. When tested on HeLa cells, the photodynamic efficacy of HA–GO/Ce6 nanohybrids was ten times that of free Ce6.

Graphene quantum dots (GQD) have attracted great interest in the biomedical field, because of their performance in bioimaging: they possess stable and strong fluorescence, along with electrical and thermal conductivity. To demonstrate the efficient delivery of GQD following receptor-mediated endocytosis to tumor tissue, Park and coworkers prepared HA-coated GQD. To explore their theranostic ability, DOX was then bound onto the surface via π–π interaction [119]. HA was anchored to the surface after derivatization with dopamine (degree of substitution 1.65%) by simple carbodiimide chemistry. The size of GQD increased, after HA anchoring, from 5–12 nm to 35–55 nm, but strong blue luminescence was observed in both cases. On CD44 + A549 cells the native toxicity of GDQ was reduced after HA-derivatization; however, compared to DOX-loaded material, there was a significant increase of cytotoxicity. After i.v. administration in mice, both native GQD and HA-modified GQD were found in the liver and kidney, as well as in tumor tissues, although double the quantity of HA-GQD accumulated in tumor tissues. 

#### 5.2.3. Fullerene

The smallest known carbon-based nanostructure, buckminsterfullerene (C_60_), has also received considerable attention in the oncological field, because it is regarded as being an excellent photosensitizer for use in photodynamic therapy. [120] Like the carbon nanostructures reported above, fullerenes are also insoluble in water and biological media and, due to their extreme hydrophobicity, form supra-aggregates limiting their photosensitivity. In recent work, Kwag *et al.* described the preparation of hyaluronated fullerenes [121] produced in the presence of lithium hydroxide, as catalyst. LiOH reacted with fullerene to break the π–π carbon bonds, after which fullerene was combined with the HA hydroxyl group, yielding various forms of carbon–oxygen conjugates (Figure 3D). 

By this method, using LMW-HA (4 kDa), nanoparticles of 30–60 nm were obtained; it is interesting that these nano conjugates exhibited NIR fluorescence, detected at 710 nm after excitation at 635 nm, although their fluorescence intensities gradually decreased as the degree of C_60_ substitution increased (from 0.05 to 0.78 C_60_ per one sugar unit of HA). After irradiation, the compounds were found to be cytotoxic on CD44 + cells. After intravenous administration to nude mice bearing HCT-116 tumors, C_60_-HA effectively accumulated in the tumor tissue, although high fluorescent intensity was also noted in the liver. 24 h after injection of C_60_-HA (0.05 C_60_ per one sugar unit of HA) and laser irradiation, the tumor volume was monitored for 7 days. Notably, significant regression in tumor volume was observed.

#### 5.2.4. Carbon Nanotubes

CNTs are graphite-like structures that are inert in nature. In terms of biomedical applications, carbon nanotubes have shown important potential in several areas, from tissue engineering to drug delivery. Regarding their applications in delivering therapeutic agents, please see a recent review [122]. Generally, there are three ways in which CNTs can be loaded with drugs for use in drug delivery. Firstly, the drug can be chemically functionalized, either permanently or via cleavable linkers, onto the surface of CNTs. Secondly, drugs that possess conjugated aromatic ring systems can be physically adsorbed onto the surface of CNTs via non-covalent π–π and hydrophobic interactions. Thirdly, some drugs can be encapsulated within the interior cavity of CNTs. As targeting or solubilizing agent, HA can be adsorbed onto the CNT surface, or may be covalently linked (either directly or after derivatization).

**Figure 4:**
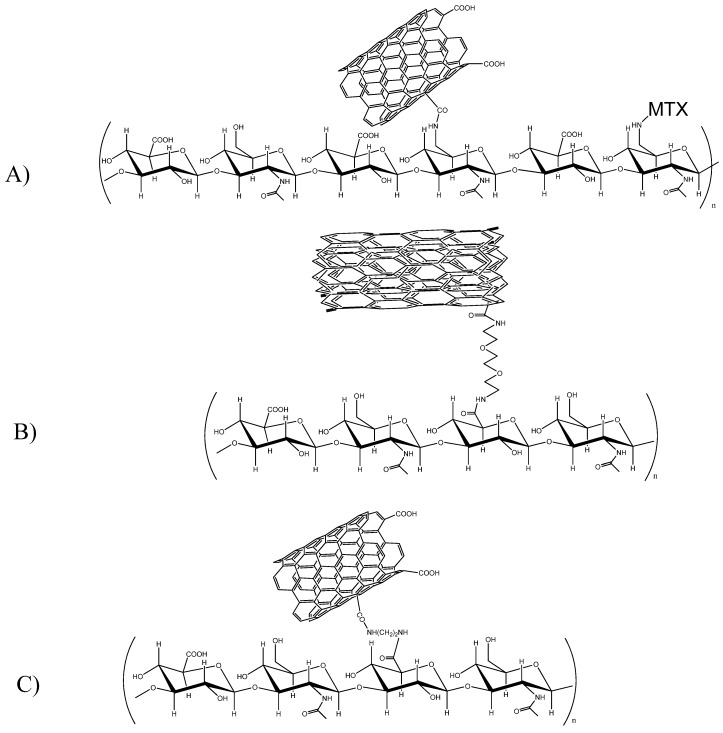
Different ways to conjugate carbon nanotubes to HA (MTX ‒ methotrexate).

A “complete covalent” approach has been presented by Marega *et al.*: HA was functionalized with amino groups at the primary hydroxyl groups of the N-acetyl-d-glucosamine moiety. Single-walled carbon nanotubes (SWCNT) are oxidized to generate carboxyl groups, using the mineral-acid-based wet process, after which the carboxylic groups are activated by reaction with NHS. Isolated ox-SWCNT-OSu is allowed to react with oligo-HA-NH_2_ as TBA salt, to form an oligo-HANHCO-ox-SWCNT conjugate. The free amino groups of the oligo-HA-NH_2_ chains are then further functionalized with the anti-inflammatory drug ibuprofen, and with the anticancer drug methotrexate [123] (Figure 4A). No *in vivo* data are yet available.

One of the most recent approaches consists of inserting cholanic-derivatized HA onto SWCNT [124]. Probe sonication was required to induce the hydrophobic interaction of cholanic acid with the SWCNT surface, and in turn disrupt the intertube van der Waals interactions. This simplified coating of HA-nanocarbons and purification. HA-SWCNTs were also chemically labeled with fluorescent dyes and a PET probe, to confirm their selective CD44 receptor uptake in cells and *in vivo* tests. Biodistribution studies on an SCC7 tumor-bearing mouse demonstrated rapid uptake and long-term accumulation of HA-SWCNTs in the tumor regions.

The same approach, without the SWCNT coating, was employed in an earlier study to generate HA-NP, composed of only HA-cholanic and a luminescence probe [125,126]. The degree of substitution (number of ethyl cholanic molecules per 100 sugar residues of HA) was controlled, and remained in the 2–10 range. A greater degree of derivatization led to a smaller size of NP. In this study, which used HMW-HA (2.5 MDa), tumor targeting predominated over liver capture, especially for the conjugate with derivatization degree 10 (size 230 nm). Liver uptake was still significant, but tumor uptake was higher with HA-NP than with the HA-polymer, due to the formulation’s prolonged blood survival. The study suggested that this high tumor targeting of HA-NPs might be due to a combination of an EPR effect, as well as to receptor-mediated endocytosis of HA-NPs.

The covalent linkage of HA onto multiwalled carbon nanotubes (MWCNT), followed by adsorption of DOX, was described by Jain [127]. As a preliminary, the functionalization required the oxidation of MWCNT, after which the carboxyl functions were interchanged with amine groups, through a sequence of thionyl chloride induced acylations (24 h) and further reaction with around 3–5-fold molar excess of 2,2'-(ethylenedioxy) bis(ethylamine). HA of different sizes (120 and 5 kDa) was tested; based on the cost of HA, and the tendency of 120 kDa HA to form meshwork around the CNTs, LMW-HA was found to be more effective (Figure 4B). DOX was loaded into CNTs at pH 7.5–8, exploiting the supramolecular π–π stacking interactions between drug and nanotubes, and the increased lipophilicity of DOX in slightly basic solutions. A DOX loading of 33% w/w was obtained. Interestingly, the biodistribution profile showed that uptake to MPS organs (liver, spleen, and lung) was lower than it was with non-HA-derivatized or amino-decorated CNTs. Furthermore, the tumor-specific accumulation of HA-targeted MWCNTs was around 5.79 and 3.18 times higher, respectively than it was with free DOX and non-HA-targeted, amino functionalized MWCNTs. Nevertheless, a single dose of 5 mg/Kg in rats with mammary tumor inducted with 7,12-dimethylbenz[α]anthracene demonstrated slightly higher antitumoral activity than that of free DOX; there was no significant difference between HA and HA-deprived DOX loading NTs. The same group then extended this approach, by comparing different drugs (DOX, PTX, methotrexate), different targeting moieties (HA, folic acid, estradiol) and PEG [128].

A similar approach was recently described by Shi *et al.* [129], in which HA-decorated SWCNT were used to deliver hematoporphyrin monomethyl ether (HMME, Hemoporfin^®^), a photodynamic therapy photosensitizer that has been in clinical trials in China since the early 1990s, to obtain a nanoparticle photodynamic therapy (PDT) agent. Moreover, photothermal therapy of CNTs in the NIR region (808 nm), and PDT of HMME in the visible light region (532 nm), were administered simultaneously to reinforce antitumor effects. After oxidation, the SWCNT was reacted with ethylenediamine and then with HA (Mw 14–20 kDa) (Figure 4C). HMME was adsorbed by sonication. A very high loading capacity (230% w/w) was observed. The anticancer activity of these compounds was tested on mouse melanoma tumor models (B16F10 cells) by intravenous administration every 2 days, and irradiation with 532/808 nm laser. Tumors in mice in the irradiated HMME-HA CNTs arm showed the slowest growth after treatment (15% *versus* saline controls).

### 5.3. Iron Oxide Nanoparticles

The field of superparamagnetic iron oxide nanoparticles (SPION) and HA conjugated gold nanoparticles has not been as widely explored as that of carbon-nanostuctures. For diagnostic purposes, SPIONs were coated with near-infrared fluorescence dye (Cy5.5)-labeled HA [130]. Physicochemical analyses of Cy5.5-HA coated SPIONs demonstrated high colloidal stability and T2 relaxivity, suggesting that they have potential as magnetic resonance probes. Furthermore, after treatment with hyaluronidase, the fluorescent signal of Cy5.5-HA-SPIONs increased considerably, due to degradation of the HA backbone. 

**Figure 5:**
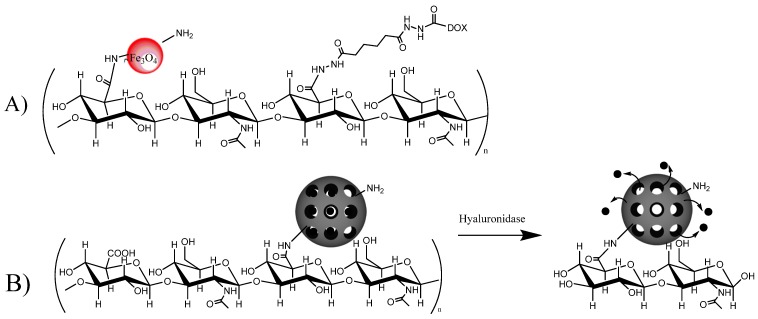
SPION (or SNP) loading HA-linked DOX (**A**); MSP and drug release after treatment with Hyaluronidase-1 (**B**).

Based on previous research on quantum dots, which showed that HA derivatives can be delivered target-specifically to liver sinusoidal cells, Hahn *et al.* described the synthesis of HA-interferon alpha gold nanoparticles for targeted treatment of the hepatitis C virus [131]. A similar approach was followed by Huang *et al.* to target iron-oxide-based magnetic nanoparticles, bearing HA on the surface, to activated macrophages [132]. The same group exploited this synthetic procedure to cover both superparamagnetic iron oxide nanoparticles [133] and silica nanoparticles (see below) with HA, and then with DOX. Nanoparticles having a solid core of 5 nm, and a hydrodynamic diameter of 114 nm, in which HA (31 kDa) accounted for 44% of the weight, were able to link DOX at a loading level of 2.1% by weight. They thereby produced a theranostic agent that combined magnetic resonance imaging of cancer cells with biocompatibility and cytotoxicity (Figure 5A). Furthermore, the DOX-HA-SPION was found to be more cytotoxic to cancer cells (including multidrug resistant lines) compared to free drug.

### 5.4. Silica Nanoparticles

Mesoporous silica nanoparticles (MSP) are now attracting attention as promising components of multimodal nanoparticle systems, owing to their straightforward synthesis, tunable pore size, large loading capacity, good chemical stability, and good biocompatibility [134]. In a recent development, MSP-nanoreservoirs derivatized on their surface with HA have been studied, with the goal of adding the further advantages of enhanced biocompatibility and CD44 targeting. MSP of 100 nm and with pores of average diameter of 2.9 were modified with 3-(2-aminoethylamino) propyltrimethoxysilane, after which HA (100 kDa) was conjugated by carbodiimide reaction [135]. HA covered the pores of MSP, and complete release of rhodamine B, used as model drug, was achieved, but only after treatment with the lysosomal enzyme hyaluronidase-1 (Figure 5B). Cytotoxicity assays of MSP-HA loading DOX (at 68.5 µg/g SiO_2_) using CD44 expressing and control cell lines, showed a small, but significant, increase in activity on the CD44 + cells. 

Core-shell silica nanoparticles (SNP) with a fluorescent core were produced by Huang and coworkers, using 3-(aminopropyl)-triethoxysilane co-polymerized with tetraethylorthosilicate in an oil–water microemulsion. A coating of HA (31 kDa), covalently linked through amide bond, was then added, and increased the SNP’s solubility and CD44 targeting. In the latest step, DOX was linked to HA using conjugation with ADH [136] (Figure 5A). The resulting HA-DOX-SNP had a diameter of 112 nm, loaded 31% w/w HA, and contained a DOX fraction of 0.6% w/w. The activity and specificity were assayed on cell lines and in 3D cell models (SKOV-3 spheroids); it was found that the HA coating significantly enhanced the SNPs’ tumor penetration ability and activity, including against DOX-resistant ovarian cancer cells, compared to free drug [137].

Using a similar approach, Durand *et al.* prepared SNP for photodynamic therapy: a core of a water-soluble photosensitizer was entrapped in the silica framework, and then a polyelectrolyte multilayer was added, composed of polylysine and HA, for surface functionalization. The cytotoxicity results demonstrated higher phototoxic efficiency of HA-SNP after three days of growth after irradiation [138].

The principal characteristics of HA- nanostructures conjugates have been summarized in Table 4.

**Table 4:** Summary of the characteristics of HA-nanostructures conjugates.

HA Mw (kDa)	Nanostructures	Conjugation chemistry	Name (or components)	DL	Particle size (nm)	*In vivo* admin.	Tumor model	Effects	Ref.
130	Quantum dot (imaging)	Amide linkage	HA-ADH	22, 35 68 ADH	7–12	SC, IV	Biodistribution	22,35% liver, 68% tissues	[106,107]
100	Carbon nanodots (amino)	Amide linkage	Amide		68	IV	Low cell toxicity, solubility	Liver targeting	[116]
5.8	Graphene oxide sheets, chlorin e6	Amide linkage	HA-ADH	115	78		HeLa cells, photodynamic	Increased activity	[118]
230	Graphene quantum dots, doxorubicin	Amide linkage	HA-dopamine	75	35–55	IV	CD44+ cells, biodistribution	High tumor conc, *in vitro* activity	[119]
4	Fullerene (C60)	Carbon–oxygen linkage		0.05 to 0.6 C60/sugar	30–60	IV	Cell phototoxicity	High tumor conc, Inhibit tumor growth	[120]
234	SWCNTs	Amide linkage	HA-cholanic + cyanine, DOTA			IV	Biodistribution	High fast tumor uptake	[124]
234	Spontaneous nanoparticle	Amide linkage	HA-cholanic, cyanine		237–424	IV	Biodistribution	Prolonged circulation, high tumor uptake	[125,126]
120 and 5	MWCN, doxorubicin	Amide linkage	Amide + 99mTc, AlexaFluor	33		IV	*In vitro, in vivo*, biodistribution, toxicity	High liver and tumor uptake	[127]
14–20	MWCN, Hemoporfin	Amide linkage		230		IV	Photodynamic therapy	Inhibit tumor growth	[129]
31	SPION, doxorubicin	Amide linkage	Amide/HA-ADH-DOX	2.1	114		Human monocytic cell line	NMR+delivery	[133]
100	MSP DOX	Amide linkage	Amide, DOX	3,7	100	IV	Human breast cancer	High tumor uptake	[135]
31	Core-shell silica nanoparticles, DOX	Amide linkage	Amide/HA-ADH-DOX	0.6	112		*In vitro* 3D model	Increased tumor penetration	[137]

SWCNT, single-walled carbon nanotubes ; MWCNT, multiwalled carbon nanotubes; SPION, superparamagnetic iron oxide nanoparticles; MSP, Mesoporous silica nanoparticles; ADH, adipic dihydrazide; HA, hyaluronic acid; DL = drug loading content, expressed as % w/w.

## 6. Conclusions

HA has been studied in considerable depth, because of its interesting physicochemical and biological properties, namely biocompatibility, biodegradability, non–toxicity, and non-immunogenicity. A further interesting characteristic is that it can easily be chemically modified, and used as a vector for actively targeting drugs or various carrier systems for cancer diagnosis and therapy. Much research has demonstrated HA’s targetability towards cancer cells overexpressing the CD44 receptor. This review has concentrated on the different chemical strategies adopted to synthesize conjugates and prepare novel delivery systems with improved performance. 

Many different chemical approaches have been used to conjugate HA to drugs, to different particulate carriers (micro and nanoparticles, liposomes, lipoplexes, etc) and to inorganic matrices. Of the numerous resulting products, one in particular, HA-PTX, known also as ONCOFID-P, which is administered locoregionally against bladder cell carcinoma, ovarian cancer and gastric tumors, is currently in phase II trials. A phase I trial has also been begun to investigate the maximum tolerated dose and safety profile following i.p. infusion, in patients affected by intraperitoneal carcinosis in ovarian, breast, stomach, bladder and colon cancers.

The possibility of conjugating HA to particulate systems may be expected to provide opportunities to target cancer cells with drugs that cannot easily be linked to HA directly. Several groups report interesting results in various *in vitro* and *in vivo* preclinical studies dealing with targeting drug-loaded nanoparticles with surface-attached HA.

Inorganic matrices (such as quantum dots, carbon nanotubes and nanodots, graphene, gold nanoparticles, iron oxide nanoparticles, and silica nanoparticles) have been studied in cancer therapy, where they have a wide range of possible applications in imaging, drug delivery, and theranostics. More recently, these systems have been modified by conjugation with HA, which gives them novel characteristics and improves their targetability; to date this approach has only been evaluated in preclinical studies. 

Few HA-drug delivery systems are yet in clinical trials. Several points must be studied and understood in depth in order to design and improve the efficacy of systems based on CD44 targeting. It is clear that several parameters, such as HA’s molecular weight, the site and degree of chemical modifications, its size and surface characteristics, can all significantly affect the interaction of HA-modified system with the target, avoiding preferential uptake by the reticuloendothelial system. In particular, in authors opinion the chemical conjugation strategies play a crucial role in the preparation, stability, *in vitro* and *in vivo* activity of HA-based conjugates. Further research into these and other parameters offer promising prospects for interesting new developments in this field.
